# Mesangial sclerosis in a patient with type 1 diabetes following simultaneous pancreas-kidney transplantation despite maintenance of normoglycemia: a case report

**DOI:** 10.1186/s12882-021-02620-7

**Published:** 2021-12-11

**Authors:** Boonphiphop Boonpheng, Jonathan E. Zuckerman, Gerald S. Lipshutz, Gabriel M. Danovitch, Angela Phelps, Michele Pena, Julie M. Yabu

**Affiliations:** 1 Department of Medicine, Division of Nephrology, David Geffen UCLA School of Medicine, 7-155 Factor Building; Mail Code 168917, Los Angeles, CA 90095 USA; 2 Department of Pathology and Laboratory Medicine, David Geffen UCLA School of Medicine, Los Angeles, USA; 3 Department of Surgery, Division of Liver and Pancreas Transplantation, David Geffen UCLA School of Medicine, Los Angeles, USA; 4 Department of Urology, David Geffen UCLA School of Medicine, Los Angeles, USA

**Keywords:** Kidney-pancreas transplant, Diabetic nephropathy, Mesangial sclerosis, Case report

## Abstract

**Background:**

Simultaneous pancreas-kidney transplantation is considered a curative treatment for type 1 diabetes complicated by end-stage kidney disease. We report herein a case of mesangial sclerosis in a patient who underwent successful kidney-pancreas transplantation despite well-controlled glucose and excellent pancreatic allograft function.

**Case presentation:**

A 76-year-old type 1 diabetic man who underwent a simultaneous pancreas-kidney transplantation 19 years prior presented with persistent nephrotic range proteinuria although creatinine was at his baseline (normal) level. Hemoglobin A1c and fasting glucose were well controlled without the use of insulin or oral antihyperglycemic agents. Serum lipase and amylase were within the reference range and there was no evidence of donor-specific antibodies. Kidney allograft biopsy was performed to evaluate proteinuria and showed diffuse capillary loop thickening and diffuse moderate to severe mesangial sclerosis resembling diabetic nephropathy.

**Conclusions:**

This case demonstrates a case of mesangial sclerosis resembling diabetic nephropathy in a patient with good glucose control after simultaneous pancreas-kidney transplantation with excellent pancreatic allograft function.

## Background

Pancreas allotransplantation is considered a curative treatment option for type 1 diabetic patients, and simultaneous pancreas-kidney transplantation (SPK) is an option for patients with kidney disease and type 1 diabetes. Limited data suggested that successful pancreas transplantation could prevent or reverse diabetic nephropathy [1–3] by re-establishing glucose homeostasis and maintenance of normoglycemia. In this report, we present a case of a patient who had mesangial sclerosis resembling diabetic nephropathy despite long-term maintenance of normoglycemia after successful pancreas-kidney transplantation.

## Case presentation

The patient is a 76-year-old type 1 diabetic man who underwent a SPK transplant 19 years prior. The donor was a 48-year-old woman with no history of diabetes and cause of death was head trauma. The donor’s final creatinine was 44.2 micromol/L (0.5 mg/dL) and urinalysis did not show proteinuria. The recipient had type 1 diabetes since age 7 complicated by mild retinopathy. Kidney disease was presumed secondary to diabetic nephropathy (no native kidney biopsy performed) which progressed slowly to end-stage kidney disease (ESKD) requiring peritoneal dialysis a year prior to transplantation. He received induction with basiliximab and had been maintained on standard triple immunosuppression with tacrolimus, mycophenolate acid and prednisone. His transplant course had been excellent without rejection episodes or major complications.

On a routine clinic visit 19 years after his transplant, urinalysis showed 2+ proteinuria without cells or casts. Urine protein-creatinine ratio ranged from 3 to 4.3 g/day on multiple repeat measurements, concordant with a 24-h urine collection, which showed 3.5 g/day of proteinuria. His baseline antihypertensive regimen included amlodipine and metoprolol. Losartan was started after proteinuria was detected. Serum creatinine remained within his baseline level (88.4–106.1 micromol/L, 1–1.2 mg/dL, eGFR 58–68 mL/min/1.73m^2^). Physical examination was notable for trace pedal edema but otherwise unremarkable. Review of previous laboratory results showed a positive dipstick proteinuria was first noted 2 years ago (17 years after transplant). Fasting glucose level has been 5–5.2 mmol/L (90–94 mg/dL) and hemoglobin A1c had been well controlled, ranging from 5.2–5.8% without the use of insulin or any other glucose lowering medications. Lipase and amylase were within reference range on two laboratory checks (amylase 88 and 91 U/L; Lipase 35 and 18 U/L). His home blood pressure was well-controlled. Body mass index was 20.5 kg/m^2^. Single antigen assay demonstrated no donor-specific antibodies. Immunosuppression was tacrolimus with levels 6.5–10 ng/mL, mycophenolic acid 360 mg twice daily and prednisone 5 mg/day.

**Fig. 1:**
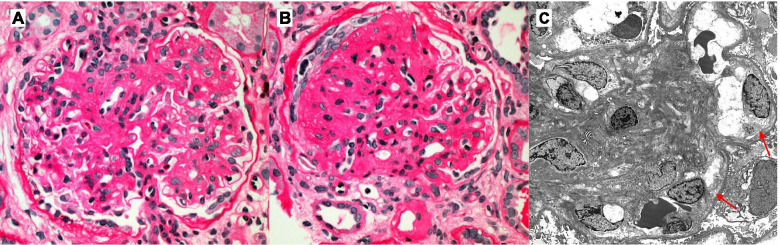
Light microscopy demonstrated (**A**) moderate to severe mesangial sclerosis as well as (**B**) peri-hilar segmental sclerosis (Periodic Acid Schiff stain; 400x). Electronic microscopic studies demonstrated (**C**) moderate to severe mesangial sclerosis and subtle very early double contour formation (arrows)

Due to new-onset nephrotic range proteinuria, kidney allograft biopsy was performed (Fig. 1). The biopsy contained 30 glomeruli of which 8 were globally sclerotic. By light microscopy, glomeruli exhibited diffuse capillary loop thickening and diffuse moderate to severe mesangial sclerosis. At least 4 glomeruli exhibited segmental sclerosis, mostly in perihilar locations. There was no glomerulitis, peritubular capillaritis, mesangial hypercellularity or amyloid type deposits. There were no necrotizing lesions or crescents. There were no capillary loop double contours. Moderate tubular atrophy and interstitial fibrosis (30–40%) was present and was focally involved by chronic inflammation. There was no tubulitis or significant inflammation within the non-scarred cortex. There was severe arteriosclerosis and intimal hyaline arteriolosclerosis. There was focal (~ 20%) immunofluorescence C4d staining of peritubular capillaries. Glomerular capillary walls and tubular basement membranes exhibited pseudo linear staining for IgG and albumin. Areas of segmental sclerosis exhibited smudgy IgM, C1q and C3 staining. There was no other significant glomerular or tubulointerstitial immunofluorescence staining. Electron microscopy demonstrated moderate to severe mesangial sclerosis and generally intact podocyte foot processes. Some glomerular capillary loops exhibited minimal segmental early basement membrane double contouring. Glomerular basement membrane thickness could not be assessed as the electron microscopy studies were performed on re-processed paraffin tissue. Peritubular capillaries showed normal basement membranes. There were no immune complex type deposits (including fibrils or microtubules) present in any location. Overall diagnostic impression favored mesangial sclerosis resembling diabetic nephropathy with secondary perihilar focal segmental sclerosis. Given the very early double contour formation on electron microscopic studies a component of chronic transplant glomerulopathy could not be excluded. No previous kidney biopsy was performed or available for comparison.

After the biopsy results, we started the patient on losartan with proteinuria decreasing in the range of 2 to 3 g/day and creatinine at baseline (1.2 mg/dl or eGFR 68 ml/min/1.73 m^2^). We also optimized blood pressure control as well as preventive measures such as monitoring lipid panel and fasting glucose levels which remained normal.

## Discussion and conclusion

We report a case of mesangial sclerosis resembling diabetic nephropathy in a type 1 diabetic patient who underwent a successful SPK without clinical or biochemical evidence of graft failure as demonstrated by excellent hemoglobin A1c, normal serum amylase/lipase and no need for insulin or oral hypoglycemic medications.

Diabetic nephropathy can recur after kidney transplantation in diabetic patients but only rarely leads to graft loss [4]. Intensive glycemic control may prevent or delay recurrence of pathologic changes of diabetic nephropathy. A randomized controlled trial [5] comparing intensive and standard glucose control in type 1 diabetic patients undergoing kidney transplantation alone showed increase in glomerular basement membrane width, mesangial matrix volume and arterial hyalinosis in both groups at 5 years but the standard group had a two-fold greater increase in mesangial matrix volume fraction on kidney biopsy compared to the intensive glucose control group. Notably even in the intensive control group, hemoglobin A1c remained above normal range (5.1–7.3%); therefore, whether pathologic lesions of diabetic nephropathy would still develop if complete normoglycemia was achieved remains unanswered.

Pancreas transplantation is considered a curative treatment of type 1 diabetes, and diabetic nephropathy lesions can possibly regress or at least be prevented after a successful SPK. This was illustrated by a study by Fioretto et al. [1] that showed significant regression of mesangial fraction volume on kidney biopsy at 10 years but not at 5 years in eight type 1 diabetic patients with biopsy-confirmed diabetic nephropathy undergoing pancreas transplantation alone. In contrast, Kim et al. [6] reported a case of diabetic nephropathy progression in a type 1 diabetic patient after successful pancreas transplantation alone (PTA). The patient had normal kidney function (eGFR 123.3 mL/min/1.73m^2^ by CKD-EPI) and only moderately increased albuminuria (174.4 mg/g urine albumin creatine ratio) prior to transplant. After pancreas transplantation, hemoglobin A1c was normalized to less than 5%. Prior to pancreas transplantation hemoglobin A1c was 11.1%. However, proteinuria continued to increase beginning 2 months post-transplant. A percutaneous kidney biopsy performed at 52 months after transplantation showed basement membrane thickening (> 500 nm by electron microscopy) and Kimmelstiel-Wilson nodules typical of diabetic nephropathy; there was no evidence of calcineurin inhibitor toxicity or other changes.

The finding in this case raise questions about the underlying mechanisms of diabetic nephropathy and whether factors other than hyperglycemia may play a role in its pathogenesis. There are case reports of biopsy-confirmed diabetic nephropathy and diabetic retinopathy in patients without diabetes even with normal glucose levels [7, 8].

Nodular glomerulosclerosis without diabetes (also known as idiopathic nodular glomerulosclerosis), characterized by mesangial matrix expansion and nodularity similar to those seen in classic diabetic nephropathy, has been described [9]. These pathologic changes have been associated with smoking and long-standing hypertension [9]. It has been hypothesized that smoking or long-standing hypertension may lead to formation of advanced glycation products, oxidative stress, abnormal renal hemodynamics and angiogenesis, contributing to pathologic changes in the kidney similar to diabetes [10]. A literature review of idiopathic nodular glomerulosclerosis by Lopez-Revuelta et al. [11] also found association of this pathologic entity with obesity and metabolic syndrome. Together, this suggests that insulin resistance even without frank diabetes may play a central role in the pathogenesis of both diabetic nephropathy and idiopathic nodular sclerosis. Our patient did not smoke tobacco and his blood pressure on anti-hypertensive medications and BMI were normal; thus, these factors do not explain the pathologic findings.

Interestingly, the biopsy demonstrated focal C4d positivity and features suspicious for very early chronic transplant glomerulopathy. While these findings do raise the possibility of some component of chronic active antibody-mediated rejection, the degree of mesangial sclerosis is significantly more advanced than would be typically seen with these relatively mild antibody-mediated changes. Furthermore, no multi-layering of peritubular capillary basement membranes was present and there was not any microvascular inflammation. However, we cannot exclude the possibility that chronic antibody mediated rejection was responsible for these biopsy changes. In addition, there was absence of donor specific antibodies or history of any prior rejection episodes that would support an ongoing immune process. Furthermore, progressive allograft dysfunction is often seen in transplant glomerulopathy, which was not seen in this patient.

Significant arteriolar hyalinosis was present. This finding can be seen with diabetic nephropathy, age, chronic hypertension, as well as with chronic calcineurin inhibitor therapy. Chronic calcineurin inhibitor toxicity could also be a factor in the changes in this biopsy including the segmental glomerulosclerosis; although, mesangial sclerosis is not generally considered to be typical features seen in this entity.

Other differential diagnoses for mesangial sclerosis include light chain deposition disease, fibrillary glomerulopathy and other esoteric deposition disease (e.g., fibronectin glomerulopathy). The immunofluorescence studies were negative which rule out light chain deposition disease, fibrillary glomerulopathy or other immune complex mediated processes. The electron microscopic studies did not reveal any other deposits or fibrils. A congo red stain was not performed; however, no other features suspicious for amyloidosis were present. In addition, a serum protein electrophoresis was negative for a monoclonal gammopathy. Of note, the electron microscopic studies were mildly limited due paraffin reprocessing artifacts.

One limitation in diagnosing abnormal glucose metabolism in routine clinical practice is that day-to-day variation in glucose may not be detected by using hemoglobin A1c. Therefore, it is possible that transient hyperglycemia might have occurred undetected in our patient, which contributed to the pathologic changes in the kidney. One case report described a patient with kidney biopsy findings compatible with diabetic nephropathy with impaired glucose tolerance but without frank diabetes but continuous glucose monitoring later revealed hyperglycemia fulfilling criteria for diabetes [12].

We report an unusual case of mesangial sclerosis resembling diabetic nephropathy in a patient with type 1 diabetes who underwent successful pancreas-kidney transplantation with excellent pancreatic allograft function, long-term maintenance of normoglycemia as demonstrated by hemoglobin A1c and insulin independence.

## Data Availability

All data generated or analyzed during this study are included in this published article.

## Abbreviations

SPK: Simultaneous pancreas-kidney transplantation
PTA: Pancreas transplantation alone
ESKD: End-stage kidney disease
eGFR: Estimated glomerular filtration rate
GBM: Glomerular basement membrane
BMI: Body-mass index
